# Selective steroid oxyfunctionalisation by CYP154C5, a bacterial cytochrome P450

**DOI:** 10.1186/1475-2859-12-95

**Published:** 2013-10-17

**Authors:** Paula Bracco, Dick B Janssen, Anett Schallmey

**Affiliations:** 1 Junior Professorship for Biocatalysis, Institute of Biotechnology, RWTH Aachen University, Worringerweg 3, Aachen, 52074, Germany; 2 Biochemical Laboratory, Groningen Biomolecular Sciences and Biotechnology Institute, University of Groningen, Nijenborgh 4, Groningen, 9747 AG, Netherlands

**Keywords:** Cytochrome P450 monooxygenase, CYP154C5, Steroid hydroxylation, Regioselective, Stereoselective, Whole-cell catalysis, *Nocardia farcinica* IFM 10152, Hydroxypropyl-β-cyclodextrin

## Abstract

**Background:**

Cytochrome P450 monooxygenases – able to regio- and stereoselectively hydroxylate non-activated carbon atoms – are important enzymes for the synthesis of valuable intermediates in the production of steroid hormones in the pharmaceutical industry. However, up to now only a few bacterial enzymes able to hydroxylate steroids have been reported. CYP154C5 from *Nocardia farcinica* IFM 10152, a bacterial P450 monooxygenase, was previously shown to convert testosterone to 16α-hydroxytestosterone. Since the hydroxylation at 16α-position is of special interest for the pharmaceutical industry, we have studied this enzyme in more detail to investigate its activity and selectivity in bioconversions of further steroids.

**Results:**

CYP154C5 was coexpressed in *Escherichia coli* together with putidaredoxin and putidaredoxin reductase from *Pseudomonas putida* as redox partners for electron transfer and applied in bioconversions of various pregnanes and androstanes [pregnenolone (**1**), dehydroepiandrosterone (**2**), progesterone (**3**), androstenedione (**4**), testosterone (**5**) and nandrolone (**6**)]. Structure elucidation of the formed products revealed an exclusive regio- and stereoselectivity of CYP154C5, always yielding the corresponding 16α-hydroxylated steroids. Application of whole cells expressing the three components, P450, Pdx and PdR, in steroid biotransformations resulted in significantly higher conversions and total turnover numbers (TTN) compared to reactions using cell-free extracts. Additionally, considerably higher substrate loads (up to 15 mM) were tolerated by the whole-cell system. Furthermore, turnover numbers (TON) were determined for the six different steroids using whole cells. Thus, testosterone was found to be the worst substrate with a TON of only 0.8 μmol substrate consumed min^-1^ μmol^-1^ CYP154C5, while progesterone and pregnenolone were converted the fastest resulting in TON of 3.3 μmol substrate consumed min^-1^ μmol^-1^ CYP154C5.

**Conclusion:**

CYP154C5 from *N. farcinica* constitutes a promising catalyst due to its high regio- and stereoselectivity in the hydroxylation of different steroids as well as its efficient expression in *E. coli* at high yields. Using this enzyme, 16α-hydroxylated steroids, which are important precursors for the synthesis of high value steroidal drugs in the pharmaceutical industry, can be selectively produced on preparative scale with TTN (μmol substrate consumed μmol^-1^ CYP154C5) exceeding 2000.

## Background

Cytochrome P450 enzymes (CYPs) form a huge superfamily of heme-containing monooxygenases present in all domains of life, where they play important roles in the detoxification of xenobiotics, drug metabolism, the assimilation of carbon sources and the formation of secondary metabolites [1]. They catalyze a number of chemically diverse reactions like hydroxylations, epoxidations, dealkylations and dehalogenations [2,3], making them very attractive catalysts for application in the synthesis of fine chemicals and pharmaceuticals. In this respect, the most important reaction is the selective oxidation of (unactivated) C-H bonds using molecular oxygen. Thus, P450 monooxygenases are applied in the pharmaceutical industry for the regio- and stereoselective hydroxylation of steroid compounds – a reaction that can be hardly achieved by chemical methods – to form highly valuable steroid hormones like glucocorticoids, mineralocorticoids and sexual hormones [2,4].

Because of their natural role in steroid synthesis, today a lot more eukaryotic CYPs (mainly mammalian enzymes) capable of steroid hydroxylation are known compared to prokaryotic ones. However, the biotechnological use of eukaryotic P450 monooxygenases is significantly hampered by the fact that they are membrane bound within the cells [5], and often show low catalytic activities. In contrast, bacterial CYPs are soluble enzymes that usually can be overexpressed to very high amounts allowing much higher productivities in biotransformations. Nevertheless, only a very limited number of known bacterial cytochrome P450 monooxygenases are able to perform selective steroid hydroxylations [6]. Despite the fact that a large number of bacterial species are reported in literature to carry out hydroxylation of various steroids [7-10], the genes coding for the respective CYPs are in most cases not known and the enzymes usually have been only poorly characterized. In a recent study, Agematu *et al*. examined a library consisting of 213 different bacterial CYPs for their ability to convert testosterone [11]. Of these 213 enzymes only 24 turned out to be active on this steroid substrate, exhibiting also different regio- and stereoselectivities. One of these active P450 monooxygenases is CYP154C5 from *Nocardia farcinica* IFM 10152. Agematu *et al.* reported that this enzyme was able to convert 100 μM testosterone (**5**) selectively into 16α-hydroxytestosterone (**11**) using whole cell catalysis with an overall conversion >10% [11]. *N. farcinica* is a human pathogen causing a disease called nocardiosis by infection of lung, central nervous system, brain and cutaneous tissues. The bacterium belongs to the phylum of *Actinobacteria* and is naturally found in soils. The genome sequence of *N. farcinica* IFM 10152 was recently determined revealing the presence of 21 genes coding for putative cytochrome P450 monooxygenases, of which two were shown to hydroxylate steroids [12]. One of them is CYP154C5, a multi-component P450 monooxygenase. For its activity, the enzyme needs additional electron transfer components delivering electrons from the cofactor NAD(P)H to the heme of the monooxygenase. Since the natural electron transfer partners of CYP154C5 are not known, electron transfer components of other CYP systems have to be used as substitutes. Thus it was shown before that putidaredoxin (Pdx) and putidaredoxin reductase (PdR) from the P450cam system of *Pseudomonas putida*[11,13] as well as the reductase domain of P450RhF (CYP116B2) from *Rhodococcus* sp. NCIMB 9784 [14] can be applied as surrogate electron transfer partners required for CYP154C5 activity.

Steroids are structurally derived from the four-ring system sterane and vary in the presence of functional groups and the oxidation state of the rings. The type, number and position of functional groups in the steroid backbone directly influence the physiological activity (such as the control of distinct aspects of cell proliferation, tissue differentiation and quorum sensing) [8]. Therefore, the regio- and stereoselective introduction of functional groups, such as hydroxylations, is important for the bioactivity of steroid drugs in a wide range of therapeutic applications, namely as anti-inflammatory, immunosuppressive, progestational, diuretic, anabolic or contraceptive agents. Today, about 300 approved steroid drugs are known and they represent the second largest category of marketed medical products next to antibiotics [8]. Current studies on microbial steroid hydroxylation involve the search for novel biocatalysts capable of performing oxyfunctionalizations at positions 7α, 9α, 11α, 11β, 16α and 17α [7,8,15]. Oxyfunctionalization at position 11β is important for anti-inflammatory steroid drugs such as cortisol, whereas 16α-hydroxylated steroids are important intermediates in the synthesis of highly active glucocorticoids [4]. Thus, the identification, cloning and biocatalytic characterization of bacterial cytochrome P450 monooxygenases able to hydroxylate steroids of different structure at the specified positions in a highly regio- and stereoselective fashion is of high importance for the pharmaceutical industry. Furthermore, a detailed analysis of optimal reaction parameters is crucial in order to develop efficient processes for the large-scale production of hydroxylated steroids using cytochrome P450s.

Based on the initial findings of Agematu *et al.*, we herein report the characterization and catalytic potential of CYP154C5 from *Nocardia farcinica* IFM 10152 for its application in regio- and stereoselective steroid hydroxylations as well as the optimization and up-scaling of respective biocatalytic reactions using different androstanes and pregnanes as substrates.

## Results

### Cloning and recombinant expression of CYP154C5

The gene of CYP154C5 was amplified from vector pKNL031_M17 from a genomic library of *N. farcinica* IFM 10152 [12] using PCR and cloned into the broad host-range vector pIT2-MCS [16]. Putidaredoxin and putidaredoxin reductase from *P. putida* DSM50198 served as electron transfer components in bioconversions [13]. For efficient coexpression of all three proteins in one host, *E. coli* C43(DE3) - a mutant of *E. coli* BL21(DE3) - was used [17]. Employing *E. coli* as a host for P450 expression and biocatalysis offers the advantage that *E. coli* naturally does not contain any endogenous P450 enzyme which could result in background activity. Protein expression was induced by IPTG addition and δ-aminolevulinic acid was added for enhanced heme synthesis. Additionally, trace element solution was used to supplement the media especially with iron. Protein production at 10 L fermentor scale with controlled oxygen supply and pH resulted in a higher expression level compared to shake flasks (0.5 L). In particular, expression of the redox partners Pdx and PdR was more efficient. The obtained specific activity of the electron transfer components (ETC activity) was in the range of 1.1 to 1.4 U/mg total soluble protein at fermentor scale in comparison to only 0.6 to 0.8 U/mg obtained in shake flasks. With regard to P450 expression, approximately 2.4 mg of CYP154C5 per g of wet cells (corresponding to 45 mg CYP154C5 per liter culture and 1.7% CYP154C5 of total soluble protein) were produced during 24 h cultivation in the fermentor.

### Steroid conversions on analytical scale: whole cells versus cell-free extract

**Figure 1:**
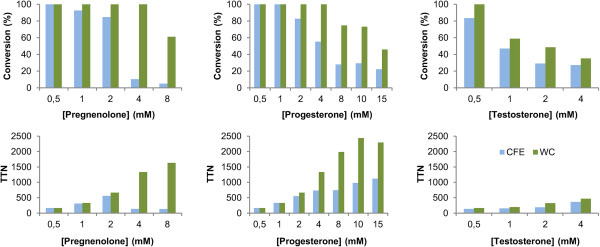
**Steroid bioconversions by whole cells (WC) and cell-free extract (CFE) of *E. coli *C43(DE3) (pIT2cyp154C5) (pACYCcamAB) employing each 3 μM CYP154C5.** Results are given as conversions (%) and total turnover numbers (TTN, μmol of substrate consumed per μmol of CYP154C5 present in the reaction mixture) obtained in biotransformations of pregnenolone (**1**), progesterone (**3**) and testosterone (**5**) using different initial substrate concentrations. All reactions were carried out in 50 mM potassium phosphate buffer pH 7.4 at 30°C for 20 h. Substrates were added from stock solutions in 36% w/v hydroxypropyl-β-cyclodextrin in water. For cofactor regeneration in reactions using CFE, 0.5 U/mL formate dehydrogenase from *Candida boidinii* and 150 mM sodium formate were employed. In reactions using whole cells cofactor regeneration was achieved by addition of 30 mM glucose.

In order to identify optimal reaction conditions for steroid transformations using CYP154C5, bioconversions employing resting whole cells (WC) or cell-free extract (CFE) of *E. coli* C43(DE3) (pIT2cyp154C5) (pACYCcamAB) were compared. Additionally, the influence of increasing concentrations of steroid substrate (0.5 to 8 mM for substrates **1**, **2**, **5** and **6**; 0.5 to 15 mM for substrates **3** and **4**) was investigated. In the whole-cell system an OD_600_ of 40 was used, which is equivalent to 3 μM CYP154C5 and an ETC activity of 9 U/mL (corresponding to a specific activity of 1.4 U/mg) as determined by analysis of cell-free extract that was obtained after cell disruption. In the case of bioconversions using CFE 3 μM P450 and 6 U/mL (0.8 U/mg total protein) ETC activity were employed. Since the three proteins were coexpressed in one host (for the whole-cell biocatalyst as well as the CFE), only the level of one of them was kept constant in the biocatalytic reactions (3 μM CYP154C5 in this case). In bioconversions using CFE efficient regeneration of the required cofactor NADH was realized by addition of a cofactor regeneration system consisting of formate dehydrogenase from *Candida boidinii* and sodium formate. In contrast, glucose was added for NADH regeneration within the *E. coli* metabolism in case of whole-cell reactions. As controls, either whole cells or CFE of *E. coli* C43(DE3) (pACYCcamAB), not expressing the cytochrome P450, were employed in steroid biotransformations. Steroid conversions were determined by GC or HPLC based on substrate depletion and total turnover numbers (TTN, μmol of substrate consumed per μmol of CYP154C5 present in the reaction mixture) were calculated. For all tested substrates application of whole cells resulted in higher conversions and higher TTN than cell-free extract (Figure 1, see Additional file 1: Figure S2 for substrates **2**, **4** and **6**). In all cases, except for testosterone (**5**), the difference in conversion and TTN between whole cells and CFE increases with increasing substrate concentration. Hence, the whole-cell biocatalytic system is especially more suitable for reactions with high initial steroid concentration. Highest TTN were obtained for progesterone (**3**, TTN = 2440) and androstenedione (**4**, TTN = 3341) for 10 and 15 mM substrate concentration, respectively, using whole cells. In contrast, TTN in steroid transformations using CFE hardly exceeded 1000. However, it has to be stressed that despite the high TTN full substrate conversion was not achieved within 20 h reaction time using high initial substrate concentrations (≥ 8 mM). In control reactions with *E. coli* C43(DE3) (pACYCcamAB) not expressing CYP154C5 no product formation was observed (Additional file 1: Figure S1).

Comparing reactions of the different substrates using whole cells, it becomes obvious that **1**, **2**, **3** and **4** are still fully converted for initial steroid concentrations of up to 4 mM, whereas conversions obtained for substrates **5** and **6** are already significantly reduced at substrate concentrations above 0.5 mM. Hence, achieved TTN for **5** and **6** are also significantly lower as compared to steroids **1**–**4**. Additionally, the influence of cell density on the performance of the whole-cell biocatalytic reactions was investigated. For that, *E. coli* cells containing CYP154C5, Pdx and PdR were resuspended to obtain OD_600_ of 20, 30, 40 and 60 and tested in bioconversions with up to 8 mM **1**, **3** and **5**. For all substrates, an optical density of 40 yielded the highest conversions (data not shown). Other than expected, product formation was not further increased in whole-cell reactions with OD_600_ of 60. This might be attributed to an insufficient oxygen input and distribution in reactions with high cell mass since oxygen availability plays an important role in monooxygenase-catalyzed reactions [18].

**Figure 2:**
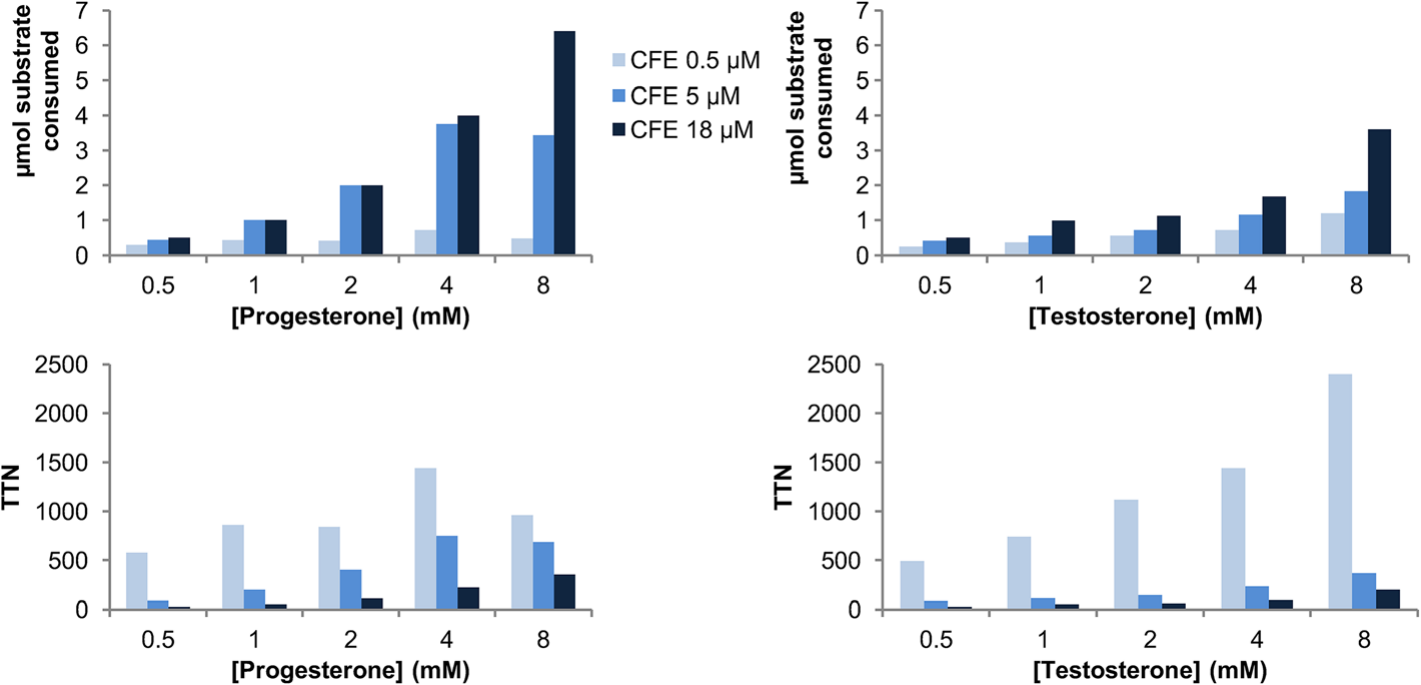
**Steroid bioconversions with cell-free extract (CFE) of *E. coli *C43(DE3) (pIT2cyp154C5) (pACYCcamAB) employing different CYP154C5 concentrations.** CFE reactions with different concentrations of progesterone (**3**) and testosterone (**5**) were performed in 50 mM potassium phosphate buffer pH 7.4 at 30°C for 24 h. Sodium formate (150 mM) and formate dehydrogenase from *Candida boidinii* (FDH, 0.5 U/mL) were used for cofactor regeneration (NADH, 50 μM). Substrates were added as stock solutions in 36% w/v hydroxypropyl-β-cyclodextrin in water.

While resting cells proved to be superior to CFE bioconversions if comparable P450 concentrations were applied, the influence of different P450 concentrations in reactions with CFE was also investigated. Thus, CFE conversions with different P450 concentrations were performed for progesterone (**3**) and testosterone (**5**) using also different substrate concentrations (from 0.5 to 8 mM). For this, concentrated CFE was diluted with different volumes of buffer in order to obtain 0.5, 5, and 18 μM final P450 concentration. The specific activity of the electron transfer components PdR and Pdx stayed constant at 0.4 U/mg total protein (corresponding to 3.2 U per μM CYP154C5). As expected, the more enzyme present in the reaction mixture the more substrate was converted (Figure 2). At the same time the obtained TTN decreased significantly with increasing enzyme concentration. Thus, highest TTN were achieved when using only 0.5 μM P450. However, the absolute amount of hydroxylated steroid that can be produced at such low enzyme concentration is still low. Thus, for a preparative-scale reaction where high product yields are aspired, application of a higher P450 concentration would be preferred. Furthermore, the addition of more NADH and formate dehydrogenase (FDH) for cofactor regeneration in the CFE reactions did not increase conversion further (data not shown). Thus, the cofactor regeneration system is not limiting the performance of the P450 system in our reactions with CFE even if high P450 concentrations are applied.

When comparing the results obtained for 18 μM CYP154C5 using CFE (Figure 2) and those obtained in whole-cell bioconversions (OD = 40) of all steroids applying 3 μM CYP154C5 (Figure 1), whole cells still outperform reactions with CFE. Similar product amounts could be achieved in whole-cell reactions, although six times lower P450 concentrations were used in comparison to CFE reactions. Hence, also significantly higher TTN can be obtained in whole-cell bioconversions (Additional file 1: Figure S3). Therefore, whole cells overexpressing the three proteins (CYP154C5, Pdx and PdR) and directly regenerating NADH within the *E. coli* metabolism proved to be more efficient for the production of hydroxylated steroids compared to CFE and were thus chosen for preparative-scale reactions.

### Steroid conversions on preparative scale: product elucidation

**Figure 3:**
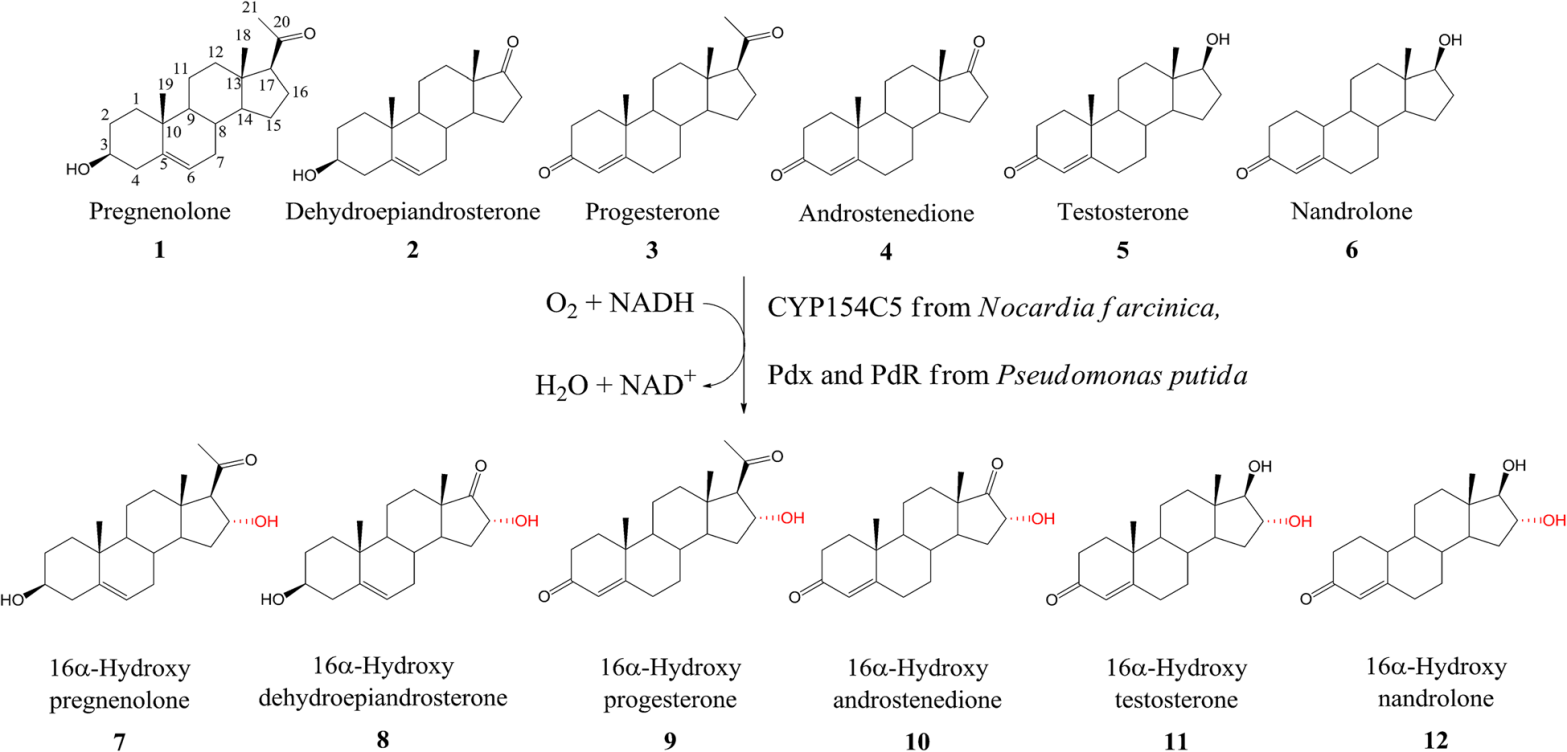
**Bioconversion of steroids 1–6 by the three component system composed of CYP154C5, Pdx and PdR.** Product structures (**7**–**12**) were determined by one and two-dimensional NMR techniques.

After investigation of important parameters for steroid bioconversion on analytical scale, best reaction conditions for each steroid were chosen for scale-up. Thus, an optical density of 40 and 2 mM substrate concentration were chosen for the whole cell steroid bioconversions at 30°C, except for substrates **3** and **5** where 4 and 1 mM were employed, respectively. In this way, sufficient amounts of products could be prepared and purified by column chromatography. Afterwards, product structures were elucidated by employing one- and two-dimensional NMR techniques in order to determine the regio- and stereoselectivity of CYP154C5 (see Additional file 1). As a result, an exceptionally high regio- and stereoselectivity was found for CYP154C5 in steroid conversions of **1**–**6** always yielding the corresponding 16α-hydroxylated products (**7**–**12**, Figure 3). Only in the case of androstenedione (**4**) and nandrolone (**6**), small amounts of a secondary product were formed. However, the amounts were so low that they could be easily removed by column chromatography purification. As mentioned before, 16α-hydroxylation of testosterone (**5**) by CYP154C5 had already been demonstrated by Agematu *et al.* on analytical scale and was now confirmed for five other steroid substrates by our NMR results [11].

Conversions and TTN achieved on preparative-scale were similar to the results of the analytical-scale biotransformations. Hence, scale up did not negatively influence the performance of the whole-cell biocatalyst. Substrates **1**, **2**, **3** and **4** were fully converted within 24 h of reaction whereas only one third and one half of the initial substrate concentration of **5** and **6**, respectively, were hydroxylated under the applied reaction conditions. Furthermore, isolated yields of the purified steroid products without optimization of the purification conditions ranged between 70-80% (40–130 mg hydroxylated steroid).

### Reaction kinetics of the resting whole-cell system

**Figure 4:**
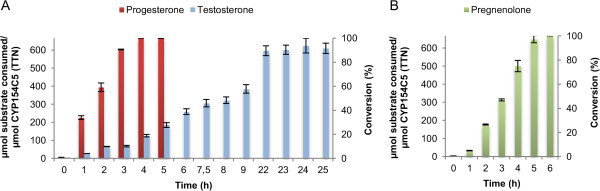
**Turnover number (TON) determination for whole cells of *E. coli *C43 (DE3) (pIT2cyp154C5) (pACYCcamAB) in steroid biotransformations.** TTN and conversion of **a)** pregnenolone (**1**) and **b)** progesterone (**3**) and testosterone (**5**) over time are given. All bioconversions were carried out in 50 mM potassium phosphate buffer pH 7.4 at 30°C with an OD_600_ of 40 and 30 mM glucose for cofactor regeneration. Substrates (2 mM) were added as stock solutions in 36% w/v hydroxypropyl-β-cyclodextrin in water. Reactions were performed in triplicate.

In order to further characterize the performance of the whole-cell system, turnover numbers (TON, μmol of substrate consumed per min per μmol CYP154C5) for all six steroid substrates were determined. In this way the efficiency of the catalytic system for the different substrates could be analyzed and compared. Bioconversions using whole cells of *E. coli* C43(DE3) (pIT2cyp154C5) (pACYCcamAB) were performed with an OD_600_ of 40 (corresponding to 3 μM CYP154C5) and 2 mM substrate concentration. As shown in Figure 4, the substrate conversion rate differs significantly depending on the applied steroid. Substrates **1**, **2**, **3** and **4** reached full conversion within the first 4–6 hours of reaction, whereas in bioconversions of **5** and **6** roughly only 50% of the substrate was converted within the first 8 hours (Figure 4). This results in space-time yields of the whole-cell system of 2.7, 3.0, 4.0, 3.6, 0.9 and 1.5 g L^-1^ d^-1^ for steroid products **7**, **8**, **9**, **10**, **11** and **12**, respectively.

Surprisingly, the conversion of some steroids, especially pregnenolone (**1**), was rather low during the first hour (Figure 4a). Thus, in order to determine the highest rates of steroid conversion by the whole-cell system, TON were calculated starting at two hours of reaction (0 and 1 h data points were not included in the calculation). As expected, **5** and **6** gave the lowest TON, approximately 1 μmol of substrate was consumed per minute per μmol of CYP154C5. In contrast, **1** and **3** gave a 3-fold higher TON in comparison to **5** and **6** (Table 1).

**Table 1:** **Calculated TON for whole-cell bioconversions of *E. coli *** **C43(DE3) (pIT2cyp154C5) (pACYCcamAB) and six different steroid substrates at 30°C**

**Substrate**	**TON (μmol substrate consumed min**^**-1**^ **μmol**^**-1 **^**CYP154C5)**
Pregnenolone (**1**)	3.28 ± 0.05
Dehydroepinadrosterone (**2**)	2.90 ± 0.17
Progesterone (**3**)	3.33 ± 0.10
Androstenedione (**4**)	2.15 ± 0.03
Testosterone (**5**)	0.82 ± 0.01
Nandrolone (**6**)	1.24 ± 0.15

Values represent the mean of triplicate measurements.

## Discussion

Industrial steroid synthesis involves sophisticated chemical and enzymatic steps including the selective incorporation of hydroxyl groups in the steroid backbone. For this reason, cytochrome P450 monooxygenases, which are able to directly hydroxylate non-activated carbon atoms, are attractive enzymes for the production of steroid hormones in the pharmaceutical industry. In the present paper, the recombinant expression and biocatalytic characterization of bacterial CYP154C5 for the regio- and stereoselective hydroxylation of different steroid compounds is reported. Since the natural electron transfer partners of this P450 are unknown, Pdx and PdR from *P. putida* were employed to supply CYP154C5 with the necessary electrons for catalysis. Using a two-plasmid system, the three different proteins, CYP154C5, Pdx and PdR, were coexpressed in soluble and active form in *E. coli* yielding good levels of CYP154C5 (approximately 2% of total protein) and sufficient activity of the electron transfer components (up to 1.4 U/mg of total protein).

Applying this three-component system in bioconversions of six different steroid substrates (**1**–**6**) we could show that CYP154C5 exhibits very high regio- and stereoselectivity not only for testosterone but also for all other tested steroids always giving 16α-hydroxylated products (**7**–**12**). Such a high specificity in the hydroxylation of various steroids carrying different functional groups and differing in the position of double bonds is remarkable since often more than one product is formed for the same steroid substrate by a P450 monooxygenase [6,11,19,20] and regioselectivities even vary with change of the steroid substrate ([6,20] and unpublished results). With CYP154C5 only in the case of **4** and **6** low quantities of a secondary product were observed in whole-cell transformations, which, however, were not formed using purified proteins. Hence, they might not be direct products of CYP154C5 catalysis. Steroid hydroxylation in 16α-position was also described in literature for a P450 monooxygenase from *Streptomyces roseochromogenes* NCIB 10984 as well as CYP123 from *Rhodococcus erythropolis* PR4 and CYP110A1 from *Nostoc* sp. PCC7120 [11,21]. However, reported steroid hydroxylation activities were rather low (e.g. 1.18 mmol progesterone converted per hour per mmol P450 for the enzyme from *S. roseochromogenes*) and further oxyfunctionalization of the product at additional positions was reported. In contrast, the here obtained TON with CYP154C5 are already by a factor of 40 to 160 higher depending on the used steroid substrate.

Comparing the efficiency of steroid bioconversions using either resting whole cells or cell-free extract of *E. coli* C43(DE3) (pIT2cyp154C5) (pACYCcamAB) clearly revealed an advantage of our whole-cell system, especially at high initial substrate concentrations. Not only significantly higher steroid concentrations (up to 15 mM) were still efficiently converted using whole cells, also substantially higher TTN and product yields could be achieved applying only low P450 concentrations. Thus, space-time yields of several grams of hydroxylated steroid per liter and day could be obtained. However, when comparing both systems it has to be taken into account that in the case of CFE reactions (i) a different cofactor regeneration system was used and (ii) the applied ETC activity was slightly lower as compared to the whole-cell system. Nevertheless we could prove that NADH regeneration by formate dehydrogenase and sodium formate in the CFE system was not limiting the overall performance of the P450 reaction. Furthermore, similar product yields for both systems could only be obtained using six times higher P450 concentration and three times higher volumetric ETC activity in CFE reactions as compared to whole cells. Thus, the slightly lower ETC activity in CFE reactions alone cannot account for the significantly reduced conversions and product yields obtained with the CFE system as compared to whole cells. Hence, the experimental results clearly prove the superior performance of our whole-cell system over reactions using CFE. In contrast, Zehentgruber et al. [6] reported that the use of crude cell extract (CCE) of recombinantly expressed CYP106A2 from *Bacillus megaterium* in *E. coli* together with bovine adrenodoxin and adrenodoxin reductase as redox partners resulted in significantly higher initial reaction rates as compared to whole cells in testosterone and progesterone bioconversions. However, the authors applied only low substrate concentrations (0.5 mM) since activity decreased significantly at higher steroid concentrations. On the other hand, the selectivity of their P450 system decreased to 80% when using CCE in comparison to whole cells due to unwanted formation of secondary hydroxylation products.

In literature, generally only low initial steroid concentrations (<1 mM) are applied in biotransformations of steroids using bacterial or eukaryotic P450 systems (whole-cell as well as isolated enzyme approaches). This might be explained by the very low solubility of steroids in aqueous solution [22], typically low rates of the applied P450s or the possible inhibitory (or even toxic) effect of steroids for microbial cells and enzymes. The low solubility of steroids is usually overcome by the use of a co-solvent such as methanol. Furthermore, the use of hydroxypropyl-β-cyclodextrin for the solubilization of steroids in aqueous solution was also reported [23-26]. Thereby, the cyclodextrin derivative offers the advantage that even high steroid concentrations are efficiently dissolved in the aqueous reaction media. In contrast, comparably high amounts of methanol, exhibiting a destabilizing effect on enzymes and cells, would be necessary to achieve the same. For potential industrial applications productivities of several grams per liter are required [8], emphasizing the need for biocatalytic systems that efficiently hydroxylate large steroid amounts with good rates and thus achieve high product titers. Our *E. coli*-based whole-cell biocatalyst containing CYP154C5, Pdx and PdR still efficiently hydroxylated steroids at concentrations of 4 mM or higher. Thus, productivities of 2–3 g/L could be achieved in case of pregnenolone (**1**), dehydroepiandrosterone (**2**), progesterone (**3**) and androstenedione (**4**). The use of hydroxypropyl-β-cyclodextrin in our reactions clearly facilitated the solubilization and thus conversion of the required steroid amounts without exhibiting any destabilizing effects on cells or enzymes. Only for testosterone (**5**) and nandrolone (**6**) much lower yields were obtained at initial steroid concentrations of 1 mM or higher. Likewise also the measured TON for the two steroids using our whole-cell system are by a factor of 2–3 lower compared to the other steroids. This might be due to a different substrate specificity of the enzyme or even an inhibitory effect of the two steroids at higher concentrations. Structurally, testosterone (**5**) and nandrolone (**6**) are very similar, both carrying a hydroxyl group at carbon 17, which might explain the similar behavior. Currently we are trying to solve the crystal structure of CYP154C5 in order to reveal structural determinants of the enzyme’s high regio- and stereoselectivity in steroid hydroxylation and to understand on a structural level the difference in reactivity of CYP154C5 towards the different steroids.

## Conclusion

Bacterial CYP154C5 from *N. farcinica* constitutes a promising catalyst due to its high regio- and stereoselectivity in the hydroxylation of various pregnanes and androstanes as well as its high-level expression in *E. coli*. Using this enzyme, 16α-hydroxylated steroids, which are important precursors in the synthesis of high value steroidal drugs such as glucocorticoids in the pharmaceutical industry, can be produced. Furthermore, an *E. coli*-based whole-cell biocatalyst – recombinantly expressing CYP154C5 together with putidaredoxin and putidaredoxin reductase as redox partners – was generated that allowed efficient conversion of the different steroid substrates even at high initial steroid concentration with the sole addition of glucose for cofactor regeneration.

## Methods

### Chemicals

Pregnenolone, testosterone, progesterone and cholesterol were purchased from Sigma-Aldrich, Germany. Dehydroepiandrosterone, androstenedione, nandrolone, 16α-hydroxypregnenolone, 16α-hydroxyprogesterone, 16α-hydroxydehydroepiandrosterone and 16α-hydroxyandrostenedione were purchased from Steraloids Inc., USA. All solvents and chemicals necessary for the experiments were obtained from Applichem, Roth or Sigma-Aldrich and used without further purification.

### Bacterial strains and plasmids

The *Escherichia coli* TOP10 strain (Invitrogen, Carlsbad, CA, USA) was used for genetic manipulations, while *E. coli* C43(DE3) (Lucigen, Middleton, WI, USA) was used for expressions.

Plasmid pACYC-Duet1 was purchased from Novagen (EMD Biosciences, San Diego, CA, USA) while the broad host-range expression vector pIT2-MCS was prepared as described elsewhere [16]. Plasmid pKNL031_M17 from a genomic library of *Nocardia farcinica* containing the gene of CYP154C5 (*nfa53110*, GenBank: NC_006361) was kindly provided by Jun Ishikawa (National Institute of Infectious Diseases, Japan). Preparation of plasmid pACYCcamAB for coexpression of putidaredoxin reductase (CamA) and putidaredoxin (CamB) from *Pseudomonas putida* was described elsewhere [16].

### Cloning of cyp154C5 from *N. farcinica*

The gene of CYP154C5 was amplified by PCR from vector pKNL031_M17 using primers NF-P-NdeI-FW: 5′-AAAAAAAACATATGAACGCCTGCCCCCATTCGG-3′ and NF-P-HindIII-RV: 5′-TTTTTTAAGCTTCACCTGCCCAGGTGGATGGG-3′. Thereby restriction sites NdeI and HindIII were introduced for subsequent cloning of *cyp154C5* into vector pIT2-MCS. The resulting vector was named pIT2cyp154C5.

### Expression of *Escherichia coli* C43(DE3) (pIT2cyp154C5) (pACYCcamAB)

*E. coli* C43(DE3) (pIT2cyp154C5) (pACYCcamAB) was used for coexpression of CYP154C5 together with CamA and CamB in one host. For that, 5 mL liquid LB media supplemented with tetracycline (10 μg/mL final concentration) and chloramphenicol (25 μg/mL final conc.) was inoculated from a frozen stock of *E. coli* C43(DE3) (pIT2cyp154C5) (pACYCcamAB). After overnight incubation at 37°C and 250 rpm, a 200 mL TB preculture supplemented with tetracycline (10 μg/mL) and chloramphenicol (25 μg/mL) was inoculated with 1% v/v of the overnight culture and incubated for 24 h under the same conditions. Finally, a 16 L fermentor containing 9.8 L of TB media supplemented with tetracycline (10 μg/mL), chloramphenicol (25 μg/mL) and 0.1% v/v trace element solution (0.50 g/L of CaCl_2_*2H_2_O, 0.18 g/L of ZnSO_4_*7H_2_O, 0.10 g/L of MnSO_4_*H_2_O, 20.10 g/L of Na_2_-EDTA, 16.70 g/L of FeCl_3_*6H_2_O, 0.16 g/L of CuSO_4_*5H_2_O and 0.18 g/L of CoCl_2_*6H_2_O) was inoculated with 2% v/v preculture. The pH was set to 7.0 and adjusted during the expression with 5 M KOH and H_3_PO_4_ solutions. Dissolved oxygen was controlled with a pO_2_ electrode. Foam formation was controlled using commercial Antifoam from Sigma. At an optical density (OD_600_) of 1 the system was induced with isopropyl-β-D-1-thiogalactopyranoside (IPTG, 200 μg/mL final conc.) and δ-aminolevulinic acid (84 μg/mL final conc.) was added as a precursor for heme synthesis. After 24 hours of expression at 30°C and 300 rpm the cells were harvested by centrifugation (4424 x *g*, 4°C for 15 min), washed with 50 mM potassium phosphate buffer pH 7.4, centrifuged again (4424 × *g*, 4°C for 15 min) and the pellets were stored at −20°C.

*E. coli* C43(DE3) (pACYCcamAB) harbouring the *camA* and *camB* gene but not the gene of CYP154C5 was used as control strain in bioconversions. In this case, expression was performed according to the procedure described for *E. coli* C43(DE3) (pIT2cyp154C5) (pACYCcamAB) in 500 mL scale with 2 modifications: only chloramphenicol (25 μg/mL) was used for selective growth and δ-aminolevulinic acid was not added during the expression.

### Enzyme assays

All assays were measured with cell lysate of *E. coli* C43(DE3) (pIT2cyp154C5) (pACYCcamAB) or *E. coli* C43(DE3) (pACYCcamAB). Thus, frozen cells were resuspended in 50 mM potassium phosphate buffer pH 7.4 in order to reach an optical density of 40 and phenylmethylsulfonyl fluoride (17 μg/mL final conc.) was added as protease inhibitor. Cell disruption was performed in an EmulsiFlex-Homogenizer (Avestin, Germany) with 5 cycles of 1500 psi. After centrifugation (17,696 x *g*, 4°C for 45 min) the resulting CFE was immediately used for the assays. CYP154C5 concentration was measured by CO-difference spectra [27] and calculated based on the maximum absorbance of CO-bound CYP154C5 at 450 nm (ϵ_450_=91 mM^-1^ cm^-1^). The activity of the electron transfer components (ETC) Pdx and PdR in CFE was determined by the cytochrome c reduction assay [28], monitoring the increase in absorbance at 550 nm (ϵ_550_=19.1 mM^-1^ cm^-1^).

### Analytical scale bioconversions

In case of whole-cell bioconversions, frozen cells of *E. coli* C43(DE3) (pIT2cyp154C5) (pACYCcamAB) overexpressing Pdx, PdR and CYP154C5 were resuspended in 50 mM potassium phosphate buffer pH 7.4 to the desired final OD_600_. All bioconversions were carried out in 1 mL scale at 30°C and 250 rpm with addition of glucose (0.54 mg/mL final conc.) for cofactor regeneration within the *E. coli* metabolism. Substrate stock solutions of steroids were prepared in 36% w/v hydroxypropyl-β-cyclodextrin in water. For substrates **3**–**6**, stocks of 100 mM were prepared whereas only 50 mM stocks could be used for substrates **1** and **2**. From the prepared stocks, the respective amounts were added to the reactions in order to achieve final steroid concentrations of 0.5 to 15 mM. Hence, the ratio between substrate and hydroxypropyl-β-cyclodextrin stayed constant in each reaction. Control reactions were carried out in parallel with *E. coli* C43(DE3) (pACYCcamAB) which contained only Pdx and PdR. After 20 hours of reaction the bioconversions and controls were extracted for subsequent HPLC and GC analysis. For that, the complete sample was extracted twice with ethyl acetate (700 μL and 500 μL) and once with chloroform (500 μL), the organic phases were combined, dried with sodium sulfate and the solvent was removed under reduced pressure.

In case of bioconversions using cell-free extract (CFE), frozen cells of *E. coli* C43(DE3) (pIT2cyp154C5) (pACYCcamAB) were resuspended in 50 mM potassium phosphate buffer pH 7.4 and phenylmethylsulfonyl fluoride (17 μg/mL final conc.) was added as protease inhibitor. Cell disruption was performed in an EmulsiFlex-Homogenizer (Avestin, Germany) with 5 cycles of 1500 psi. After centrifugation (17,696 x *g*, 4°C for 45 min) the resulting CFE was immediately used for bioconversions. In 1 mL scale bioconversions, NADH (36 μg/mL final conc.) was used as electron donor, sodium formate (10 mg/mL final conc.) and formate dehydrogenase from *Candida boidinii* (Sigma Aldrich, 0.5 U/mL final activity) were added as NADH regeneration system in biocatalyses. Steroid substrates were also added as stock solutions in 36% w/v hydroxypropyl-β-cyclodextrin in water as described for whole-cell reactions. After 24 hours of reaction at 30°C and 250 rpm, the bioconversions and controls were extracted as described for the whole-cell system.

### Preparative scale bioconversions and product purification

Preparative-scale bioconversions were carried out in 100–200 ml of 50 mM potassium phosphate buffer pH 7.4 using resting whole cells of *E. coli* C43(DE3) (pIT2cyp154C5) (pACYCcamAB) (from frozen stock) at 30°C and 250 rpm, with the addition of glucose (0.54 mg/mL) in shake flasks. In all cases, resting cells with an optical density of 40 were used which is equivalent to approximately 3 μM CYP154C5 and an ETC activity of 1.1 U/mg of total protein, as determined by CO-difference spectra and cytochrome c assay, respectively. Substrate stock solutions were prepared in 36% w/v hydroxypropyl-β-cyclodextrin in water. In general 2 mM substrate concentration was used, except for progesterone (**3**) and testosterone (**5**) where 4 and 1 mM were applied, respectively. After 22–24 h of reaction, the complete reaction volume was extracted twice with ethyl acetate (50 and 40 mL) and once with chloroform (30 mL), the organic phases were combined, dried with sodium sulfate and the solvent removed under reduced pressure. Hydroxylated steroid products were afterwards purified by silica gel column chromatography (silica gel 60; 0.2-0.5 mm; Roth) with a gradient of ethyl acetate in n-heptane to pure ethyl acetate (starting with ethyl acetate / n-heptane ratios of 6:4 for pregnenolone (**1**), 4:6 for dehydroepiandrosterone (**2**), 9:1 for progesterone (**3**) and testosterone (**5**), 7:3 for androstenedione (**4**) and 95:5 for nandrolone (**6**)) as mobile phase.

### Turnover numbers

Frozen cells of *E. coli* C43(DE3) (pIT2cyp154C5) (pACYCcamAB) were resuspended in 50 mM potassium phosphate buffer pH 7.4 to an optical density (OD_600_) of 40. This cell density was equivalent to a P450 concentration of 3 μM and a specific activity of the ETC of 0.7 U/mg of total protein, as determined by CO-difference spectra and cytochrome c assay, respectively. All bioconversions were carried out at 25 mL scale in shake flasks for 26 h at 30°C and 250 rpm with addition of 30 mM glucose for cofactor regeneration. Substrate stock solutions were prepared in 36% w/v hydroxypropyl-β-cyclodextrin in water. In all cases 2 mM initial steroid concentration was used. During bioconversions, samples were taken every hour for subsequent HPLC or GC analysis. For that, 1 mL of reaction was extracted and the sample prepared for HPLC or GC analysis as previously described. Turnover numbers were calculated based on substrate consumption and for the period of time where the highest substrate consumption rate was observed (generally between 60 and 240 minutes of bioconversion, except for testosterone (**5**) and nandrolone (**6**) were the period between 180 and 360 minutes was used). All bioconversions were done in triplicate.

### GC, GC-MS, HPLC and NMR analyses

In the case of pregnenolone (**1**) and dehydroepiandrosterone (**2**) the solid residues were redissolved in chloroform containing 30 mM cholesterol as internal standard. The samples were analyzed on a GC2010 gas chromatograph (Shimadzu) equipped with an OPTIMA 17ms column (Macherey-Nagel, Germany) with a linear gradient starting at 250°C and heating with 10°C/min until 300°C. Injector and detector temperature were set to 350 and 300°C respectively. Substrates and products were detected by FID. Both substrates, pregnenolone (**1**) and dehydroepiandrosterone (**2**), eluted at a retention time of 9.69 min and 7.94 min respectively, whereas their products eluted at 9.27 (**7**) and 10.18 min (**8**) respectively.

The dried residues of bioconversions with progesterone (**3**), androstenedione (**4**), testosterone (**5**) and nandrolone (**6**) were dissolved in acetonitrile: water (60:40) and injected on a high performance liquid chromatograph (HPLC, Beckmann Coulter) equipped with a Nucleosil 100–5 C18, 250 x 4,5 mm column (CS Chromatography Service, Germany) at 50°C. Acetonitrile: water (60:40) was used as mobile phase with a flow rate of 1 mL/min. Detection of substrates and their respective products was performed by UV absorbance at 254 (**5** and **11**), 230 (**3** and **9**) and 245 nm (**4**, **6**, **10** and **12**). The substrates eluted at 9.42 (**3**), 6.68 (**4**), 7.73 (**5**), and 5.93 (**6**) min, whereas the main products were detected at 4.95 (**9**), 4.47 (**10**), 4.12 (**11**), and 3.95 (**12**) min. Secondary products of androstenedione (**4**) and nandrolone (**6**) eluted at 5.68 and 5.22 min, respectively. In all cases conversions were calculated based on substrate consumption.

Preliminary product identification was performed with a gas chromatograph - mass spectrometer (GC-MS-QP2010S, Shimadzu, Germany) equipped with an OPTIMA 17ms (products **8**, **9**, **11** and **12**) or Supreme 5ms (products **7** and **10**) column (Macherey-Nagel, Germany) with a linear gradient starting at 250°C and heating with 10°C/min until 300°C. Injector and interface temperature were set to 300°C. For products **7**, **10** and **11**, derivatization using *N*-methyl-*N*-(trimethylsilyl) trifluoroacetamide (MSTFA activated I, Sigma-Aldrich) was necessary in order to facilitate GC-MS analysis.

Structure elucidation of formed products (**7**–**11**) was performed by ^1^H, ^13^C, COSY and HSQC NMR analysis on a Bruker AV400 instrument (^1^H-NMR 400 MHz and ^13^C-NMR 100 MHz). Measurement of product standards and structure elucidation of product **12** was performed by ^1^H, ^13^C, COSY, HSQC and NOESY NMR on a Bruker AV600 (^1^H-NMR 600 MHz and ^13^C-NMR 150 MHz). In all cases deuterated chloroform was used as solvent with TMS as internal standard except for product **7** were deuterated DMSO with TMS was used. Chemical shifts (δ) are given in ppm and coupling constant (*J*) in Hz (see Additional file 1).

## Abbreviations

CCE: Crude cell extract; CFE: Cell free extract; CYP: Cytochrome P450 monooxygenase; ETC: Electron transfer components; FDH: Formate dehydrogenase; IPTG: Isopropyl-β-D-1-thiogalactopyranoside; NADH: Nicotinamide adenine dinucleotide, reduced form; OD600: Optical density at 600 nm; PdR: Putidaredoxin reductase; Pdx: Putidaredoxin; TON: Turnover number; TTN: Total turnover number; WC: Whole cells.

## Competing interests

The authors declare that they have no competing interests.

## Authors’ contributions

AS cloned the gene of CYP154C5 and established optimal expression conditions. PB performed all other practical work. DJ helped in interpreting the results and participated in writing the manuscript. AS made substantial contributions to the design of experiments, analysis of results and participated in writing the manuscript. All authors read the manuscript and gave final approval of the version to be published.

## Supplementary Material

Additional file 1: Figure S1 Representative HPLC chromatograms of steroid bioconversions using *E. coli* C43(DE3) (pIT2cyp154C5) (pACYCcamAB) and *E. coli* C43(DE3) (pACYCcamAB) as control. **Figure S2**. Comparison of whole cells (WC) and cell-free extract (CFE) of *E. coli* C43(DE3) (pIT2cyp154C5) (pACYCcamAB) in steroid bioconversions each containing 3 μM CYP154C5; **Figure S3**. Comparison of TTN achieved in steroid bioconversions by *E. coli* C43(DE3) (pIT2cyp154C5) (pACYCcamAB) using cell-free extract containing 18 μM CYP154C5 and whole cells containing 3 μM CYP154C5 (OD_600_ = 40). Furthermore, a detailed description of product structure elucidation by GC-MS and NMR analysis is given. Click here for file
