# Supplementary material for: Selective steroid oxyfunctionalisation by CYP154C5, a bacterial cytochrome P450
Source: Microb Cell Fact. 2013 Oct 17;12:95. doi: 10.1186/1475-2859-12-95 (PMC4015549; doi:10.1186/1475-2859-12-95)
Supplement: Additional file 1: Figure S1 — Representative HPLC chromatograms of steroid bioconversions using E. coli C43(DE3) (pIT2cyp154C5) (pACYCcamAB) and E. coli C43(DE3) (pACYCcamAB) as control. Figure S2. Comparison of whole cells (WC) and cell-free extract (CFE) of E. coli C43(DE3) (pIT2cyp154C5) (pACYCcamAB) in steroid bioconversions each containing 3 μM CYP154C5; Figure S3. Comparison of TTN achieved in steroid bioconversions by E. coli C43(DE3) (pIT2cyp154C5) (pACYCcamAB) using cell-free extract containing 18 μM CYP154C5 and whole cells containing 3 μM CYP154C5 (OD600 = 40). Furthermore, a detailed description of product structure elucidation by GC-MS and NMR analysis is given. [file 1475-2859-12-95-S1.docx]

Supplementary data

**Selective steroid oxyfunctionalisation by CYP154C5, a bacterial cytochrome P450**

**Paula Bracco^1^, Dick B. Janssen^2^, Anett Schallmey^1*^**

^1^ Junior Professorship for Biocatalysis, Institute of Biotechnology, RWTH Aachen University, Worringerweg 3, 52074 Aachen, Germany

^2^ Biochemical Laboratory, Groningen Biomolecular Sciences and Biotechnology Institute, University of Groningen, Nijenborgh 4, 9747 AG Groningen, The Netherlands

^*^ Corresponding author

Email addresses:

AS: a.schallmey@biotec.rwth-aachen.de

PB: p.bracco@biotec.rwth-aachen.de

DBJ: d.b.janssen@rug.nl

**Steroid conversions on analytical scale: control reactions**

**
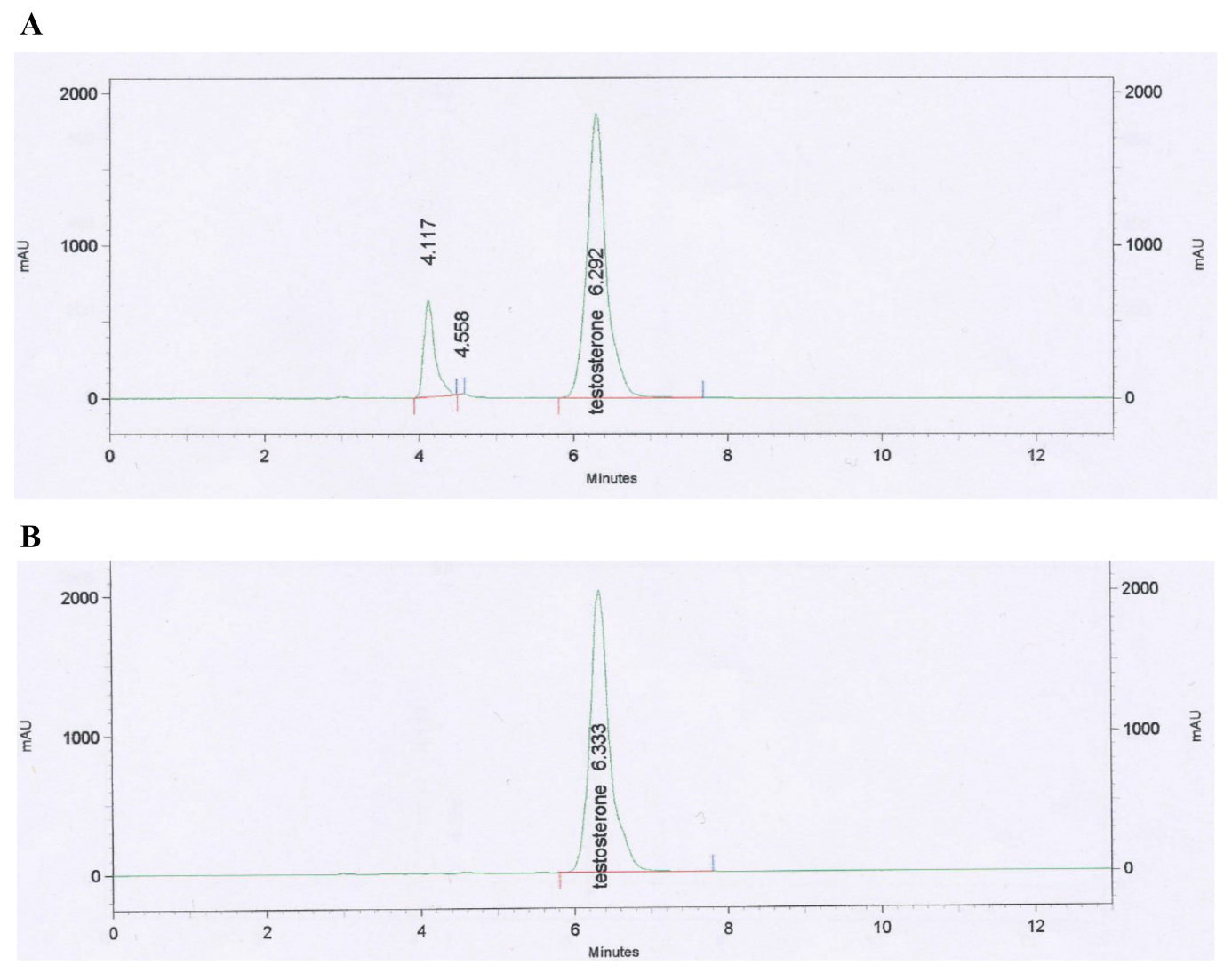
**

**Figure S1. Representative HPLC chromatograms of steroid bioconversions using *E. coli* C43(DE3) (pIT2cyp154C5) (pACYCcamAB) and *E. coli* C43(DE3) (pACYCcamAB) (control).** Shown are chromatograms of testosterone (**5**) conversions. Using *E. coli* containing CYP154C5, Pdx and PdR (**A**) product formation can be observed at 4.1 min while no product is formed in control reactions using *E. coli* only containing Pdx and PdR (**B**).

**Steroid conversions on analytical scale: whole cells versus cell-free extract**

**
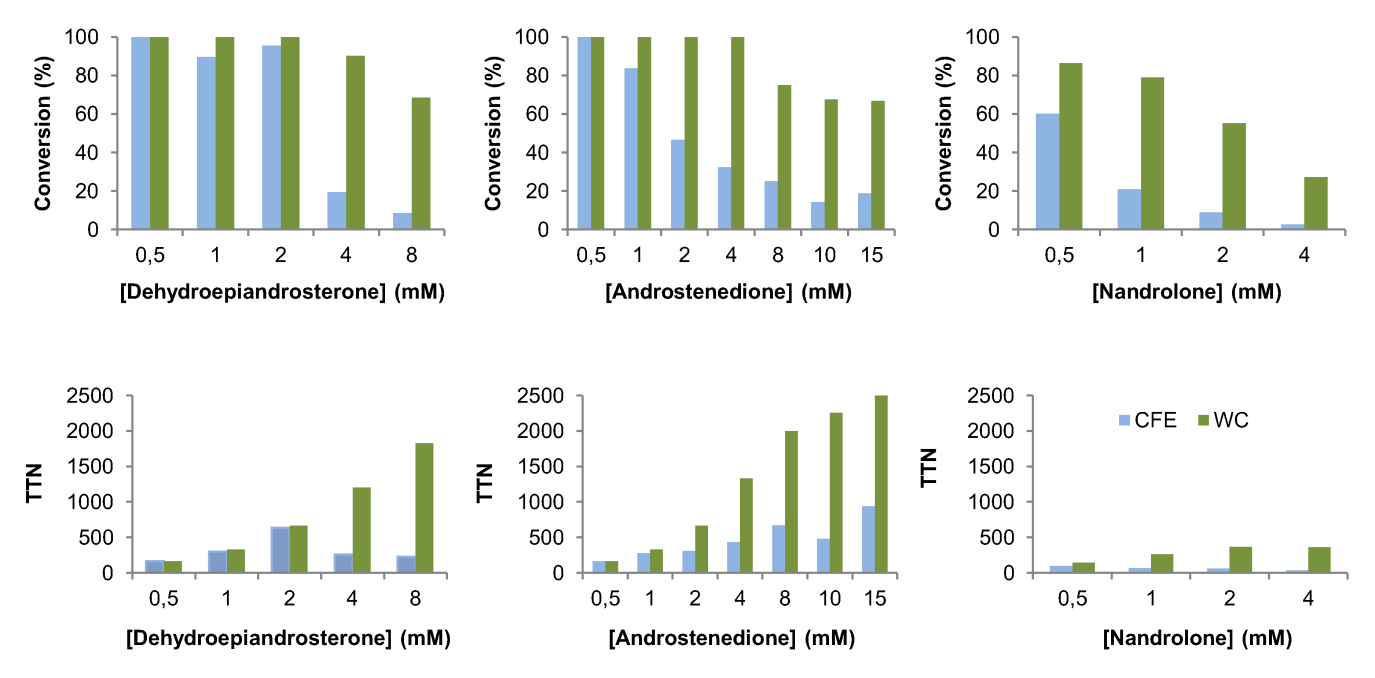
**

**Figure S2. Comparison of whole cells (WC) and cell-free extract (CFE) of *E. coli* C43(DE3) (pIT2cyp154C5) (pACYCcamAB) in steroid bioconversions each containing 3 µM CYP154C5.**
Results are given as conversions (%) and total turnover numbers (TTN, µmol of substrate consumed per µmol of CYP154C5 present in the reaction) obtained in biotransformations of dehydroepiandrosterone (**2**), androstenedione (**4**) and nandrolone (**6**) using different initial substrate concentrations. All reactions were carried out in 50 mM potassium phosphate buffer pH 7.4 at 30°C for 20 h. Substrates were added as stock solutions in 36% w/v hydroxypropyl β-cyclodextrin in water. For cofactor regeneration in reactions using CFE, 0.5 U/ml formate dehydrogenase from *Candida boidinii* and 150 mM sodium formate were employed. In reactions using whole cells cofactor regeneration was achieved by addition of 30 mM glucose.


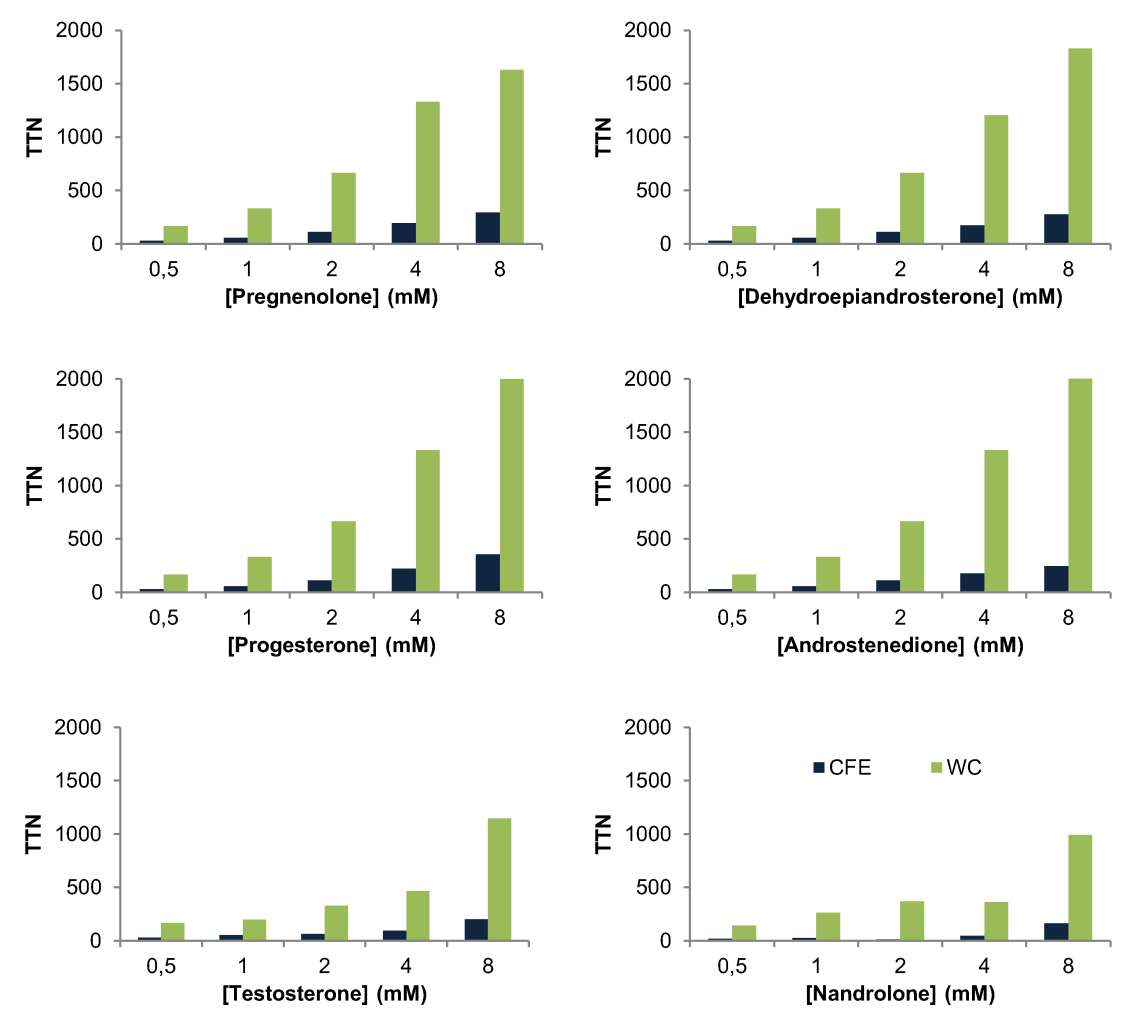


**Figure S3. Comparison of TTN achieved in steroid bioconversions by *E. coli* C43(DE3) (pIT2cyp154C5) (pACYCcamAB) using cell-free extract containing 18 µM CYP154C5 and whole cells containing 3 µM CYP154C5 (OD_600_ = 40).**
CFE reactions with different concentrations of pregnenolone (**1**), dehydroepiandrostendione (**2**), progesterone (**3**), androstenedione (**4**), testosterone (**5**) and nandrolone (**6**) were performed in 50 mM potassium phosphate buffer pH 7.4 at 30°C for 24 h. Sodium formate (150 mM) and formate dehydrogenase from *Candida boidinii* (FDH, 0.5 U/mL) were used for cofactor regeneration (NADH, 50 µM). Whole cell reactions were carried out in 50 mM potassium phosphate buffer pH 7.4 at 30°C for 20 h with addition of glucose (30 mM) for cofactor regeneration. Substrates were added as stock solutions in 36% w/v hydroxypropyl β-cyclodextrin in water.

**Product elucidation by GC-MS and NMR analysis**

**GC-MS measurement**

Preliminary product identification was performed with a gas chromatograph - mass spectrometer (GC-MS-QP2010S, Shimadzu, Germany) equipped with an OPTIMA 17ms (products **8**, **9**, **11** and **12**) or Supreme 5ms (products **7** and **10**) column (Macherey-Nagel, Germany) with a linear gradient starting at 250°C and heating with 10°C/min until 300°C. Injector and ion source temperature were set to 300 and 200°C respectively. For products **7**, **10** and **11** derivatization using N-methyl-N-(trimethylsilyl) trifluoroacetamide **(**MSTFA activated I, Sigma-Aldrich) was necessary in order to facilitate GC-MS analysis. Thus, after extraction and solvent removal, the solid residue was dissolved in 100 µL MSTFA and incubated at 65°C for 20 min before injection.

GC-MS analysis of all steroid products indicated the addition of an oxygen atom to the respective substrate.

**NMR measurement**

Structure elucidation of formed products (**7**-**11**) was performed by ^1^H, ^13^C, COSY and HSQC NMR analysis on a Bruker AV400 instrument (^1^H-NMR 400MHz and ^13^C-NMR 100MHz). Measurement of product standards and structure elucidation of product **12** was performed by ^1^H, ^13^C, COSY, HSQC and NOESY NMR on a Bruker AV600 (^1^H-NMR 600MHz and ^13^C-NMR 150MHz). In all cases deuterated chloroform was used as solvent with TMS as internal standard except for product **7** where deuterated DMSO with TMS was used. Chemical shifts (δ) are given in ppm and coupling constant (*J*) in Hz. In all cases, except for product **12**, standards were purchased and therefore their 1D and 2D NMR spectra directly compared with the NMR spectra of the CYP154C5 products. Thus, only the detailed structure elucidation of product **12** is explained in section 2.3.

**Structure elucidation**

In order to identify the position and orientation of hydroxylation in products **7**-**12,** 1- and 2-dimensional NMR data (^1^H, ^13^C, COSY, HSQC and NOESY) were analyzed and compared with the respective data of 16α-hydroxylated standards. For products **7**, **9**, **11** and **12,** the coupling constant (*J*) between 16β-H and 17α-H was the key to confirm the orientation (α and/or β) of hydroxylation to be 16α. In contrast, for products **8** and **10** the *J_Hβ15-Hβ16_* was analyzed (Table 1).

**Table S1. Chemical shifts (δ) and coupling constants (*J*) determined from ^1^H-NMR and ^13^C-NMR data of steroid products 7-12 formed by CYP154C5.**
Corresponding data of the standards are shown in brackets.

| Product | 16-C δ_C_ (ppm) | 16-H δ_H_ (ppm) | *J_Hβ15-Hβ16_* (Hz) | *J_Hβ16-Hα17_* (Hz) |
| --- | --- | --- | --- | --- |
| (**7**) 16α-hydroxypregnenolone | 72.5 (71.0) | 4.79 (5.53) |  | 6.8 (6.5) |
| (**8**) 16α-hydroxydehydroepiandrosterone | 71.3 (71.3) | 4.38 (4.40) | 7.8 (8.4) |  |
| (**9**) 16α-hydroxyprogesterone | 70.9 (73.7) | 4.78 (4.87) |  | 7.0 (6.5) |
| (**10**) 16α-hydroxyandrostenedione | 70.2 (71.2) | 4.33 (4.41) | 7.9 (8.3) |  |
| (**11**) 16α-hydroxytestosterone^a^ |  | 4.09 |  | 6.4 |
| (**12**) 16α-hydroxynandrolone^a^ | 78.3 | 4.15 | multiplet |  |

^a^ no standards purchased

Structure elucidation of product **12**.

As a first step, the ^1^H-NMR spectra of product and substrate were compared. The triplet signal at 3.77 ppm in the substrate spectrum corresponding to 17α-H, changed to a doublet at 3.52 ppm in the product ^1^H-NMR spectrum. This is the first indication of hydroxylation at position 16-C, since a hydroxyl group cannot be introduced at the other neighboring carbon (position 13-C). Furthermore, in the HSQC spectrum of **12** (Figure S3A), the proton signal at 4.15 ppm is coupling with a carbon signal at 89.7 ppm. These chemical shifts indicate the presence of a hydroxyl group at the mentioned carbon. Therefore the proton signal is assigned as 16-H. Additionally, the COSY spectrum (Figure S3B) shows a correlation between 16-H and 17α-H, and 16-H with 15β-H, confirming once more hydroxylation at position 16-C. In the NOESY spectrum (Figure S3C) coupling between 16-H and 17α-H is not observed whereas the coupling between 16-H and 15β-H is still present, indicating that 16-H is actually 16β-H. Hence, it can be concluded that CYP154C5 selectively hydroxylates nandrolone (**6**) at position 16 with α-orientation.

**
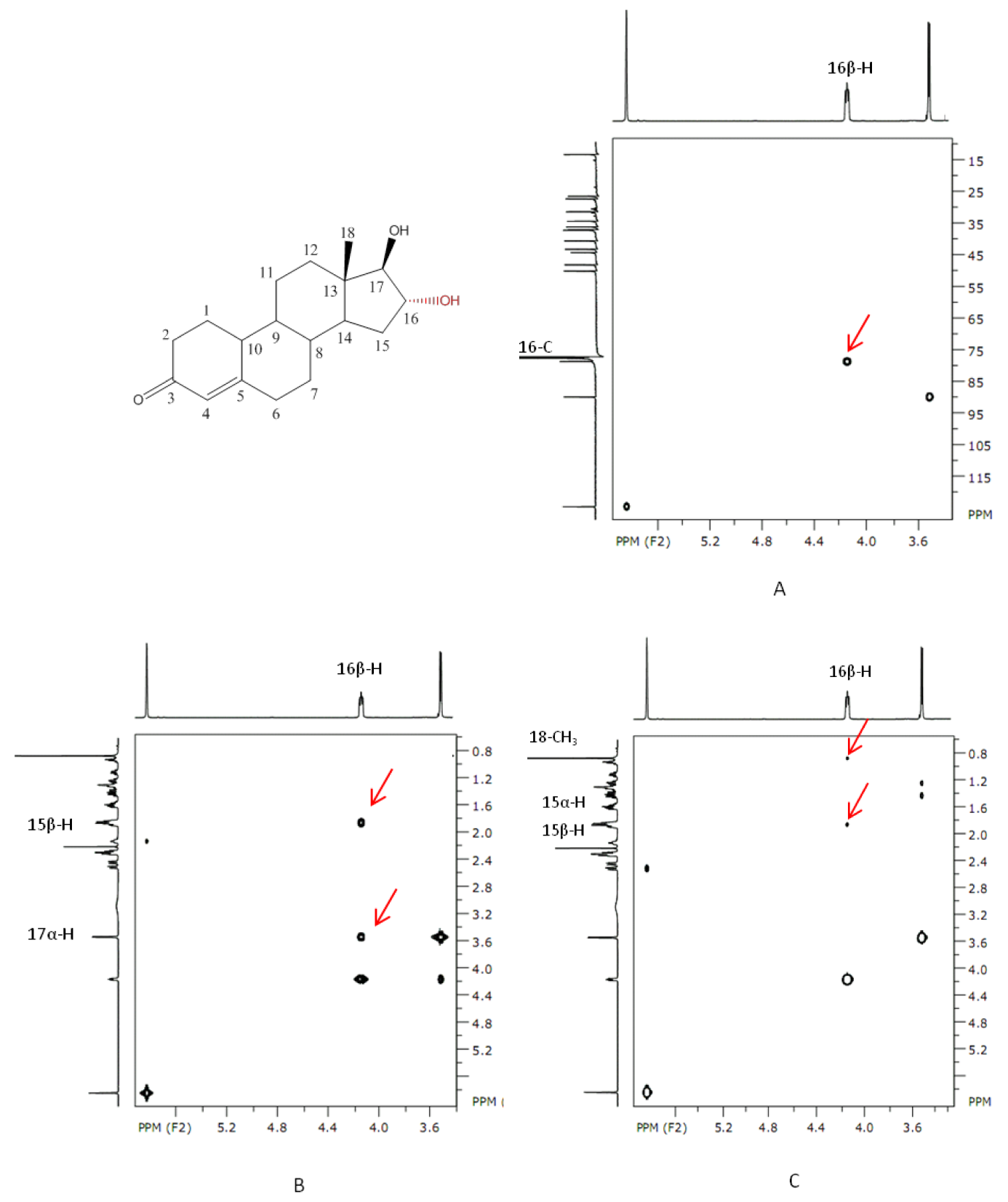
**

**Figure S4. Structure elucidation of product** **12**.
A) HSQC shows the correlation between 16β-H and the respective carbon 16-C, B) COSY shows the coupling between 16β-H with 15β-H and 17α-H , C) NOESY confirms the α-hydroxylation where 16β-H couples with 18-CH_3_ and 15β-H but not with 15α-H.

**Experimental data of obtained products**

***16α-hydroxy-pregnenolone* (*7*)**

^1^H-NMR (400 MHz, DMSO): δ 5.27 (1H, d, *J*= 4.7, 6-H), 4.70 (1H, d, *J=* 4.9, 20-OH), 4.62 (1H, d, *J=* 4.5, 3-OH), 4.52 (1H, m, 16β-H), 3.26 (1H, m, 3α-H), 2.43 (1H, d, *J*= 6.6, 17α-H), 2.19-2.03 (5H, m, 4αβ-H and 21-Me), 1.90 (2H, m, 7-H and 12-H), 1.77 (1H, d, *J*= 13.5, 1-H), 1.68 (1H, d, *J=* 12.0, 2-H), 1.60-1.28 (9H, m, 2-H, 7-H, 8-H, 12-H, 14-H and 15-H), 1.06-0.88 (5H, m, 1-H, 14-H and 19-Me), 0.54 (3H, s, 18-Me). 13C-NMR (100 MHz, DMSO): δ 208.0 (C20), 141.3(C5), 120.2 (C6), 73.0 (C17), 70.4 (C16), 69.9 (C3), 53.7 (C14), 49.5 (C9), 44.2 (C13), 42.2 (C4), 38.0 (C12), 36.8 (C1), 36.1 (C10), 35.6 (C15), 31.7 (C7), 31.4 (C8), 31.2 (C2), 31.0 (C21), 20.2 (C11), 19.1 (C19) and 14.1 (C18). ^1^H-NMR and ^13^C NMR data are consistent with data obtained for the respective standard.

Molecular weight of derivatised product: 551,5 g/mol ; GC-MS: Retention time:16.9 min. M^+^: 550 ; *m/z* (%): 550 (1), 549 (1), 533 (4), 458 (6), 231 (85), 147 (15), 131 (12), 117 (33), 73 (100).

***16α-hydroxydehydroepiandrosterone (8)***

^1^H-NMR (400 MHz, CDCl_3_): δ 5.37 (1H, d, *J=* 5.3. 6-H), 4.38 (1H, d, *J=* 7.8, 16β-H), 3.54 (1H, m, 3α-H), 2.37-2.20 (2H, m,4α-H, 4β-H), 2.07 (1H, m, 7α-H), 2.02-1.80 (5H, m, 1β-H, 2α-H, 15α-H, 15β-H), 1.73-1.33 (9H, m, 7β-H, 8β-H, 11α-H, 2β-H, 11β-H, 12β-H, 12α-H), 1.16-0.94 (8H, m, 1α-H, 9α-H), 1.92 (5H, m), 1.04 (3H, s, 18-CH_3_), 0.99 (3H, s, 19-CH_3_). ^13^C-NMR (100 MHz, CDCl_3_): δ 140.0 (5-C), 120.9 (6-C), 71.6 (3-C), 71.3 (16-C), 50.1 (9-C), 48.6 (14-C), 47.4 (13-C), 42.2 (4-C), 37.1 (1-C), 36.6 (10-C), 31.5 (2-C), 31.5 (8-C), 31.2 (7-C), 30.6 (12-C), 30.4 (15-C), 20.0 (11-C), 19.4 (19-C), 13.9 (18-C). ^1^H-NMR and ^13^C-NMR data are consistent with data obtained for the respective standard.

Molecular weight: 304.4 g/mol; GC-MS: Retention time: 8.5 min. M^+^: 304; *m/z* (%): 304 (68), 286 (39), 271 (29), 214 (39), 199 (79), 91 (91), 79 (78), 55 (71), 41 (100).

***16α-hydroxyprogesterone (9)***

^1^H-NMR (400 MHz, CDCl_3_): δ 5.67 (1H, s, 4-H), 4.78 (1H, t, *J*= 7.0, 16β-H), 2.48 (1H, d, *J*= 6.5, 17-H), 2.42-2.19 (4H, m, 2α-H, 2β-H, 6α-H, 6β-H), 2.11 (3H, s, 21-Me), 1.11 (3H, s, 19-Me), 2.01-1.91 (2H, m, 1β-H, 12β-H), 1.81-1.44 (9H, m, 1α-H, 7β-H, 8-H, 11α-H, 12α-H, 14-H, 15α-H, 15β-H), 1.37 (1H, dq, *J_q_* = 12.7, *J_d_* = 4.2, 11β-H), 1.11 (3H, s, 19-Me), 0.61 (3H, s, 19-Me). ^13^C-NMR (100 MHz, CDCl_3_): δ 123.0 (C_4_), 72.5 (C_17_), 70.9 (C_16_), 52.7 (C_14_), 52.5 (C_9_), 37.5 (C_13_), 34.6(C_12_), 34.2 (C_15_), 34.1 (C_1_), 32.9 (C_6_), 31.7 (C_2_), 30.7(C_21_), 30.7 (C_7_), 19.6 (C_11_), 16.3 (C_19_), 13.4 (C_18_). ^1^H-NMR and ^13^C NMR data are consistent with data obtained for the respective standard.

Molecular weight: 330.5 g/ mol GC-MS: Retention time: 15.8 min. M^+^: 330. *m/z* (%): 330 (1), 312 (11), 297 (6), 269 (10), 231 (26), 100 (32), 91 (25), 55 (23), 43 (100).

***16α-hydroxyandrostenedione (10)***

^1^NMR (400 MHz, CDCl_3_): δ 5.69 (1H, s, 4-H), 4.33 (1H, d, *J=* 7.9, 16β-H), 2.45-2.23 (5H, m, 2αH, 2βH, 6αH, 6βH), 2.01-1.89 (2H, m, 1βH, 15βH), 1.90 -1.76 (3H, m, 7βH, 12βH, 15αH), 1.71-1.60 (3H, m, 1αH, 8βH, 11αH), 1.52-1.27 (4H, m, 11βH, 12αH, 14αH), 1.14 (3H, s, 19-CH_3_), 1.05 (1H, dq, *J_q_* = 12.3, *J_d_* = 4.5, 6αH), 0.95 (4H, m, 9αH, 18-CH_3_). ^13^C-NMR (100 MHz, CDCl_3_): δ 217.7 (17-C), 198.2 (3-C), 168.9 (5-C), 123.3 (4-C), 70.2 (16-C), 52.3 (9-C), 46.7 (14-C), 46.4 (13-C), 37.6 (10-C), 34.6 (8-C), 34.1 (1-C), 32.9 (2-C), 31.4 (6-C), 30.0 (12-C), 29.5 (7-C), 29.4 (15-C), 18.9 (11-C), 16.4 (19-C), 13.0 (18-C). ^1^H-NMR and ^13^C NMR data are consistent with data obtained for the respective standard.

Molecular weight of derivatized product: 521 g/ mol, GC-MS: Retention time: 16.4 min. M^+^: 521; *m/z* (%): 521 (0.2), 518 (7), 503 (13), 245 (2), 147 (7), 129 (2), 75 (9), 73 (100), 45 (7).

***16α-hydroxytestosterone (11)***

^1^H-NMR (400 MHz, CDCl_3_): δ 5.67 (1H, s, 4-H), 4.09 (1H, t, *J=* 6.4, 16β-H), 3.45 (1H, d, J= 5.5, 17-H), 1.12 (3H, s, 19-Me), 0.75 (3H, s, 18-Me). NMR data was consistent with previously published data [1]. As already described [2], CYP154C5 hydroxylates **5** regio- and enantioselectively at position C-16 obtaining 16-α-hydroxytestosterone.

Molecular weight of derivatized product 523 g/mol; Retention time: 16.3 min. M^+^: 523; *m/z* (%): 523 (6), 521 (43), 520 (100), 430 (4), 325 (6), 197 (5), 147 (9), 73 (94), 45 (5).

***16α-hydroxynandrolone (12)***

^1^H-NMR (600 MHz, CDCl_3_): δ 5.85 (1H, s, 4-H), 4.15 (1H, m, 16β-H), 3.53 (1H, d, *J=* 5.74, 17-H), 2.49 (1H, dt, *J_d_=* 14.8, *J_t_=* 3.0, 6β-H), 2.42 (1H, m, 2-H), 2.31-2.23 (3H, m, 1-H, 2-H, 6α-H), 2.10 (1H, m, 9-H), 1.88-1.78 (4H, m, 7α-H, 11β-H, 12β-H, 15β-H), 1.61-1.50 (2H, m, 1-H, 15α-H), 1.43-1.16 (6H, m, 8β-H, 11α-H, 12α-H, 14α-H), 1.08 (1H, dq, *J_q_=* 13.5, *J_d_=* 4.0, 7β-H), 0.89 (1H, m, 9α-H), 0.84 (3H, s, 18-CH_3_). ^13^C-NMR (150 MHz, CDCl_3_): δ 200.2 (3-C), 166.8 (5-C), 124.7 (4-C), 89.7 (17-C), 78.3 (16-C), 49.5 (9-C), 47.5 (14-C), 43.6 (13-C), 42.6 (10-C), 39.9 (8-C), 36.5 (12-C), 36.3 (2-C), 35.4 (6-C), 33.6 (15-C), 30.6 (7-C), 26.5 (1-C), 25.7 (11-C), 12.2 (18-C).

Molecular weight: 290.4 g/ mol, GC-MS: Retention time: 10.7 min, M^+^: 290, *m/z* (%): 290 (100), 261 (15), 213 (20), 138 (50), 91 (39), 79 (36), 67 (46), 55 (51), 41 (64).

**References**

1. Kirk DN, Toms HC, Douglas C, White KA, Smith KE, Latif S, Hubbard RWP: **A survey of the high-field ^1^H NMR spectra of the steroid hormones, their hydroxylated derivatives, and related compounds**. *J Chem Soc, Perkin Trans 2* 1990:1567–1594.

2. Agematu H, Matsumoto N, Fujii Y, Kabumoto H, Doi S, Machida K, Ishikawa J, Arisawa A: **Hydroxylation of testosterone by bacterial cytochromes P450 using the *Escherichia coli* expression system**. *Bioscience, Biotechnology, and Biochemistry* 2006, **70**:307–311.
